# A double-blind placebo-controlled randomized trial of oral saffron in the treatment of rheumatoid arthritis 

**DOI:** 10.22038/AJP.2020.17280

**Published:** 2021

**Authors:** Maryam Sahebari, Hossein Heidari, Shima Nabavi, Mandana Khodashahi, Zahra Rezaieyazdi, Maliheh Dadgarmoghaddam, Hossein Hosseinzaheh, Shaghayegh Abbasi, Kamila Hashemzadeh

**Affiliations:** 1 *Rheumatic Diseases Research Center, Mashhad University of Medical Sciences, Mashhad, Iran*; 2 *Community Medicine Department,* * faculty of Medicine, Mashhad University of Medical Sciences, Mashhad, Iran*; 3 *Department of Pharmacodynamics and Toxicology, School of Pharmacy, Mashhad University of Medical Sciences, Mashhad, Iran*; 4 *Pharmaceutical Research Center, Pharmaceutical Technology Institute, Mashhad University of Medical Sciences, Mashhad, Iran*

**Keywords:** Crocus sativus L. Iridaceae family, Saffron, Rheumatoid arthritis, Clinical trial, RCT

## Abstract

**Objective:**

Recently, saffron (*Crocus sativus* L. from the Iridaceae family) has been characterized by its antioxidant, anti-inflammatory and analgesic effects. This study aimed to evaluate the effect of saffron on disease activity in patients with rheumatoid arthritis (RA).

**Materials and Methods:**

This is a double-blind, placebo-controlled, randomized clinical trial (RCT) performed on 55 newly- diagnosed RA patients without previous treatment, who were randomly divided into intervention (included 28 cases) and control groups (consisted of 27 individuals). Standard therapy including prednisolone, oral methotrexate, folic acid, vitamin D, calcium, and alendronate, was administered similarly in both groups. Patients received a 100 mg saffron pill/day (pure saffron powder) or placebo besides the standard protocol. The placebo had the same shape as the saffron pills. Follow up of DAS28ESR disease activity score was done on the 30th, 45th and 90^th^ day of the study.

**Results:**

There was no difference between the intervention and control groups regarding to the DAS28ESR at the end of the study. However, a significant decrease in DAS28-ESR was observed in each group compared to the first visit (p=0.001). The results also showed no significant difference in the incidence of side effects in both groups.

**Conclusion:**

In summary, patients who received pure saffron pills (100 mg/day) in addition to standard therapy did not have a significant difference in improvement of disease activity from the patients on standard therapy.

## Introduction

Rheumatoid arthritis (RA) is an autoimmune polyarthritis presented by considerable proliferation of synovial tissue, joint swelling, cartilage destruction, and ultimately, joint destruction in the movable joints (Kasper and Harrison, 2005 ▶; Moghimi et al., 2014b ▶). If the disease is left untreated, it will be accompanied with severe complications and disability and it can reduce the survival rate and quality of life (Hamidiet al., 2020b ▶). The prevalence of the disease was estimated to be 0.5-1% in the world, and females are three times more likely to be diagnosed with RA (NG et al., 2006b ▶; Tobón et al., 2010b ▶). Although, the etiology of this disease is unknown, epidemiological studies have presented a combination of various environmental and genetic factors associated with RA (Edwards and Cooper, 2006a ▶; Molina and Shoenfeld, 2005b ▶; Padyukov et al., 2004 ▶). Autoimmunity in the pathways of innate and adaptive immunity, cytokine production and autoantibody formation is involved in the pathogenesis of RA. The most important cytokines are IL-1β, IL-6 and TNF-α (Benucci et al., 2012 ▶; Duesterdieck-Zellmer et al., 2012b ▶; Hreggvidsdottir et al., 2014a ▶). Various treatment strategies have been proposed to treat RA in recent decades, including non-steroidal anti-inflammatory drugs (NSAIDs), glucocorticoids, and DMARDs (Disease Modifying Anti-Rheumatic Drugs), and growing evidence of biological drugs is being reported (Bolhassani, 2014a ▶; Fransen, 2004b ▶). A recent review study suggested that herbal products can have significant therapeutic effects in the treatment of certain diseases, such as diabetes, cancer, and neurodegenerative disorders. This review article provided a complete collection of studies on humans and animals that have shown significant effects of saffron on autoimmune disorders or other inflammatory diseases (Korani et al., 2019 ▶).

Saffron (*Crocus sativus* L. from the Iridaceae family) is a potent anti-inflammatory and antioxidant herb and its most effective ingredient, including crocin, crocetin and safranal, have important therapeutic properties in traditional medicine (Abdullaev and Espinosa-Aguirre, 2004a ▶; Amin and Hosseinzadeh, 2015 ▶; Javadi et al., 2013a ▶; Schmidt et al., 2007 ▶). This medical herb is widely cultivated in the countries of the Middle East and Eastern Mediterranean such as Iran, India and Greece (Ghorbani, 2008b ▶; Schmidt et al., 2007 ▶). Several studies confirmed that saffron has anti-inflammatory, antisclerotic, anti-lipid, analgesic, anticoagulant, anti-tumor and anti-cancer properties. Moreover, it plays a fundamental therapeutic role in digestive disorders (Amini and Hosseinzadeh, 2012b ▶; Amin and Hosseinzadeh, 2015b ▶; Bolhassani et al., 2014a ▶; Hosseinzadeh, 2014a ▶; Hosseinzadeh et al., 2009b ▶; Zamani Taghizadeh Rabe et al., 2015b ▶). The anti-inflammatory effect of saffron on deactivation of free radicals and its anticancer properties have been well documented; these effects are mostly the result of biological and antioxidant activity of crocin in reducing free radicals and xanthine oxidase (Hsu et al., 1999b ▶; Nair et al., 1995b ▶). Likewise, crocin has potent anti-inflammatory effects on inflammatory diseases, RA for instance, which was shown in animal studies (Rathore et al., 2015b ▶; Sahebari et al., 2011 ▶; ZamaniTaghizadehRabe et al., 2015b ▶). In 2020, a case-control study showed that saffron significantly reduced the number of painful and swollen joints, as well as the severity of pain and disease activity (Hamidi et al., 2020 ▶).

 This study aimed to determine the effect of saffron, accompanied by standard treatment, on reducing the activity of RA according to Disease activity score DAS28-ESR.

## Materials and Methods

**Figure 1:**
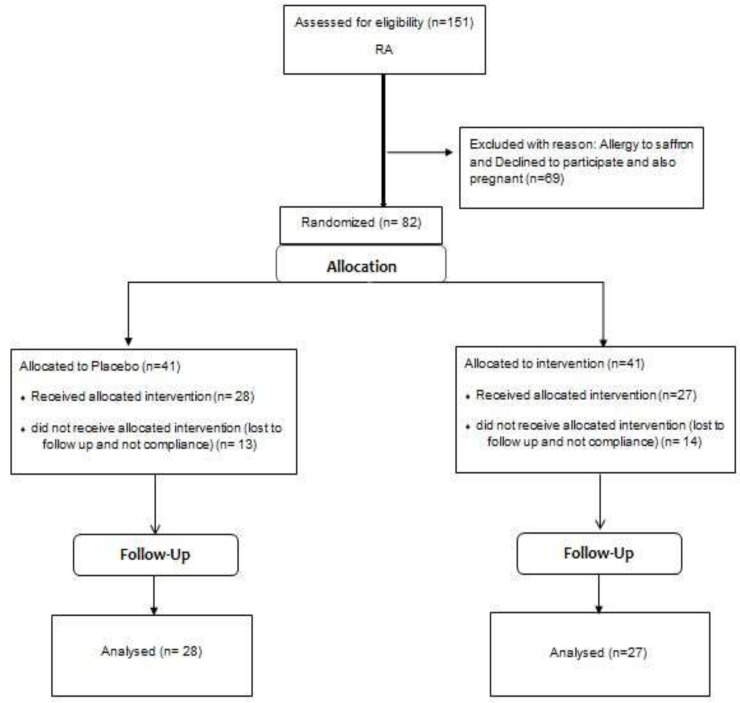
CONSORT for reporting randomized clinical trials

This is a double-blind, placebo-controlled, clinical trial performed on 55 patients with RA, who referred to Rheumatic Diseases Research Center (RDRC) of Mashhad University of Medical Sciences. In this pilot study, we determined a sample size by considering the prevalence of RA in the study population and we added 10% of its full predictable prevalence (Hertzog, 2008 ▶). At the beginning, 41 patients in each group were included in the study, but at the end, 28 patients in the intervention group and 27 patients in the placebo group were analyzed (Figure 1). Fourteen patients in the intervention group and 14 patients in the control group were excluded during the first month due to non-compliance or loss to follow-up. Informed consent was obtained from patients who were selected based on inclusion criteria (age over 18 year olds, newly diagnosed RA patients who did not receive any treatment, and having 6 out of 10 ACR/EULAR 2010 Criteria for RA) )American College Of Rheumatology/ European league Against Rheumatism). The standard of disease activity was DAS28-ESR (Disease Activity Score28-ESR). HAQ-DI (health assessment Questionnaire-disability index), VAS (visual analog scale), and Pain score (PS) and physical function questionnaires which are the important parts of HAQ-DI, were used to evaluate and compare patients’ improvement in the treatment and control groups. The reliability and validity of these standard questionnaires in Persian have been reviewed and proven in previous studies (Bruce and Fries, 2009a ▶;Kay2012 ▶; Van et al.,2013 ▶; Nazary-Moghadam etal., 2017 ▶;Rastmanesh et al., 2010a ▶).

Exclusion criteria included pregnancy and lactation, common allergy to saffron, liver or kidney disease (Renal failure GFR<80 ml/min), malignancy or active infection or psychiatric illness. This pilot study was registered in the IRCT (Iranian Registry of Clinical Trials) system (IRCT2014071218453N1).

Newly diagnosed RA patients were randomly assigned to "Standard therapy plus Saffron pills" (intervention or case) and "Standard therapy plus Placebo pills” (placebo or control) groups. The randomization method in this study included a random number table. Different rheumatologists referred patients to Rheumatic Diseases Research center (RDRC), and patients were randomized (to randomly received A or B pills) by an internal medicine specialist. Finally, a separate rheumatologist who was blind to the randomization process examined patients.

Pills containing pure saffron powder were made of saffron flower purchased from Saharkhiz Saffron Factory; in the Faculty of Pharmacy of Mashhad University. The placebo pills were prepared with the mentioned additives as saffron essence and yellow food color. After extracting saffron powder, saffron pills containing 100 mg pure saffron and additives such as starch, lactose monohydrate, starch sodium glycolate, PVP K30 were produced. The blinding method was such that A and B labels were given to the drugs in packages of one-month pill case to the patients. The physician who prescribed the packages and the physician, who examined the patients and followed up their symptoms, were blind to the drug and placebo. At the end of the study and after statistical analysis, the identity of the group A and B treatment was asked from the manufacturer. There was a special phone number for patients' follow up, besides emphasizing the use of medication; it created an easy accessibility for asking about probable complications. If a patient was going to be excluded from the study due to complications or disinclination, his/her information was recorded. According to previous human studies, a saffron dose under 400/mg produced no significant side effect (Gout et al., 2010b ▶; Modaghegh et al., 2008 ▶); so, we chose 100 mg/day in the present study. Standard therapy, which included 5 mg prednisolone/day, 7.5 mg oral methotrexate/week, 5 mg folic acid/week, 400 IU vitamin D and 800 mg calcium/day, and 70mg alendronate/week, was administered similarly to both groups. The use of any supplements such as antioxidants was banned in both groups. Any changes in the therapeutic dose of basic drugs were reported in a checklist. 

Patients were followed up for a period of 3 months (on days 30, 45 and 90). In addition, physicians called the patients every month, and asked them about any side effects or problems they may have faced following intake of the pills. A rheumatologist and an internist examined the patients to determine the disease activity by the questionnaires that mentioned before. Treatment side effects and laboratory data were recorded simultaneously in each visit. In addition to disease activity score and quality of life indices, other laboratory tests recorded at each visit (Table 1) included erythrocyte sedimentation rate (ESR), C reactive protein (CRP), Anti-cyclic citrullinated peptide (Anti-CCP), white blood cell count (WBC), hemoglobin (Hb), platelet (PLT), prothrombin time( PT), blood urea nitrogen (BUN), creatinine (cr), Aspartate transaminase ( AST), alanine transaminase (ALT), and alkaline phosphatase ( ALP).

**Statistical analysis**

The demographic data and clinical observations in both groups were analyzed using SPSS 22. Descriptive statistical methods including central indicators, dispersion and frequency distribution were used to describe the subjects' data. Given the sample size score which was lower than 100, we used Shapiro –Wilks test. Most of the main statistical data were distributed normally. For others Mann–Whitney U test was used. 

**Table 1:** Demographic and laboratory data of intervention and control groups at the base line

**p value**	**Placebo Group** **(n=27)**	**Intervention Group** **(n=28)**	**Variables**
0.39*	50.80±9.55	48.43±14.69	Age (year)
0.61**	78%	70.7%	Gender (Female)
0.19**	53.8%	31.8%	CRP (Positive)
0.10*	12.58±2.28	12.61±0.95	PT
0.65*	23.09±9.48	24.17±10.43	BUN (mg/dl)
0.35*	0.94±0.19	0.90±0.19	Cr (mg/dl)
0.76*	340.25±331	484.61±333	WBC (/mcL×x10^9^)
0.18*	12.88±1.93	16.03±17.17	Hb (gr/dL)
0.67*	273.16±86.67	274.70±58.05	PLT (/mcL×10^9^)
0.79*	19.32±5.77	19.86±5.85	AST (IU/L)
0.38*	20.34±9.83	22.07±7.38	ALT (IU/L)
0.29*	198.79±58.64	212.84±54.95	ALP (IU/L)
0.35**	81.6%	89.2%	Anti-CCP (Positive)
			

DAS28-ESR: disease activity score28 joints with ESR, VAS: Visual analog scale, HAQ-DI: Health assessment questionnaire-Disability index. *p value for independent T test and **p value for Chi square and Fisher exact test.

Independent T-test was used to compare the effects of quantitative indices between the two groups. Chi-Square and Fisher exact test were used to examine non-quantitative variables between two groups. Repeated measure ANOVA was used to examine the trend of changes in the indexes over the time intervals (repetitive p). In all tests, a p value<0.05 was considered significant. The Committee on Organizational Ethics at Mashhad University of Medical Sciences approved this research (IR.MUMS.REC.1393.77). 

## Results

In this study, from 151 newly diagnosed RA patients, 82 individuals were selected and assigned to intervention (n=41) and control (n=41) groups with a mean age of 49.32±12.37 years with female predominance (74.39%). Moreover, 27 participants refused to continue the study (no compliance=19, and poor drug compliance and slight side effects=8) during the first month of the study, with no significant difference between the two groups (p=0.6). Overall, 55 patients continued the treatment course (Figure 1).

**Figure 2:**
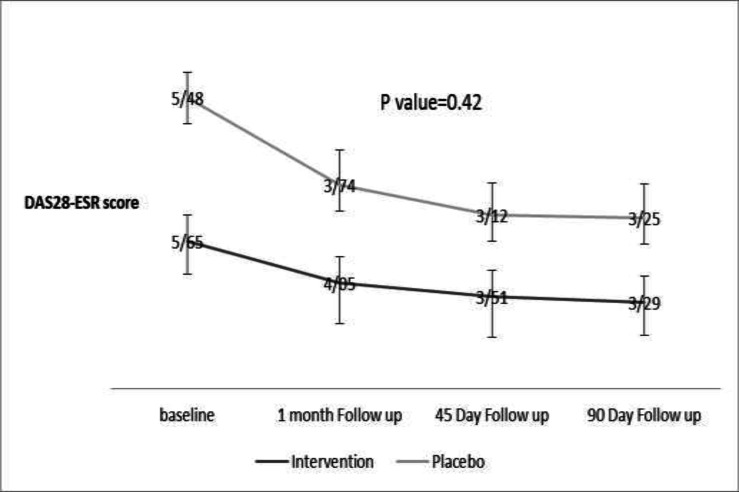
Changes in Disease activity score 28 joints -ESR (DAS28-ESR) in the two groups over the study period

Tables 1 to 3 indicate no significant difference between the two groups regarding the demographic, disease activity score, quality of life indices, clinical, laboratory data, and drug regimen at the beginning of the study. Saffron side effects that are presented in Table 4. Side effects did not have any significant difference between the two groups; even diarrhea and jaundice were not seen in our patients. Investigation of the effects of saffron pills on the improvement of DAS28ERS and quality of life questionnaires, which are presented in Table 2 and 3, showed no significant difference in the indicators of RA disease activity and quality of life between the two groups in each visit and at the end of the study. However, the trend of reduction in DAS28-ESR, VAS, poor physical function and pain score was significant in each group of the study after three-month (Table 2). Besides, drug dosage in both groups did not significantly changed over the study period (Table-2). Figure 2 projects fluctuations of DAS28-ESR in both groups during the study over the three visits. As it shows, there was not any significant difference according to DAS28ESR reduction between intervention and placebo groups. Furthermore, the survey in the need of intra-articular injection showed no difference between the groups (p=0.71).

Other laboratory parameters that mentioned in the Table 1 did not show any significant difference during and at the end of the study compared to the baseline values. Additionally, those parameters showed no difference between the groups in each visit and at the end of the study, (p values were not shown).

**Table 2:** Comparison of RA activity, quality of life and drug dosage in each group of the intervention and controls during and at the end of the study

P value**††††**	90 day follow up	P value**†††**	45 day follow up	P value**††**	1 month follow up	P value**†**	Base line	Groups	Indices
**0.331**	17.01±13.06	0.107	13.73±9.50	0.475	16.22±12.17	0.85	25.85±18.55	Intervention	ESR (mm/hr)
	17.0±12.97		20.60±15.47		20.40±15.51		34.44±19.33	Placebo	
**0.659**	3.92±4.51	0.869	4.11±5.70	0.711	5.25±6.87	0.886	11.14±7.83	Intervention	No. Tender joint
	3.52±4.16		4.0±4.69		6±5.83		10.36±6.16	Placebo	
**0.882**	2.96±3.3	0.862	3.14±4.43	0.934	4.51±5.76	0.125	14.55±22.91	Intervention	No .Swollen joint
	3.04±3.71		3.32±3.02		4.69±5.65		7.80±5.97	Placebo	
**0.179**	3.33±1.36	0.562	3.12±1.55	0.562	3.74±1.57	0.085	5.48±1.26	Intervention	DAS28-ESR
	3.34±1.18		3.59±1.21		4.07±1.39		5.66±1	Placebo	
**0.976**	22.96±20.90	0.408	22.59±21.76	0.408	38.14±25.27	0.886	62.51±29.30	Intervention	VAS
	15.20±15.57		24.40±21.22		34.40±24.16		66.80±27.19	Placebo	
**0.126**	0.74±0.6	0.685	0.83±0.67	0.737	0.94±0.64	0.942	1.36±0.67	Intervention	HAQ-DI
	0.52±0.41		0.68±0.64		0.72±0.67		1.48±0.69	Placebo	
**0.047**	0.85±0.75	0.533	0.71±0.53	0.343	1.21±0.85	0.452	2.07±0.77	Intervention	Pain Score
	0.52±0.42		0.87±0.75		0.80±0.75		2.27±0.88	Placebo	
**0.228**	2.22±1.84	0.249	3.70±7.78	0.460	3.48±2.04	0.429	5.87±2.10	Intervention	Physical function
	1.68±1.74				4.84±9.63		6.28±1.96	Placebo	

†p value trend for repeated measure test (ANOVA). ††p value between groups for independent T test.

**Table 3:** Comparison of the indices of RA disease activity and quality of life between intervention and control groups at baseline, 1 month, and 45 and 90 day follow up

**p value** **††**	**p value** **†**	**90 day follow up**	**45 day follow up**	**1 month follow up**	**Base line**	**Groups**	**Indices**
0.74	0.001	17.01±13.06	13.73±9.50	16.22±12.17	25.85±18.55	Intervention	ESR (mm/hr)
		17.0±12.97	20.60±15.47	20.40±15.51	34.44±19.33	Placebo	
0.91	0.001	3.92±4.51	4.11±5.70	5.25±6.87	11.14±7.83	Intervention	No. Tender joint
		3.52±4.16	4.0±4.69	6±5.83	10.36±6.16	Placebo	
0.30	0.005	2.96±3.3	3.14±4.43	4.51±5.76	14.55±22.91	Intervention	No. Swollen joint
		3.04±3.71	3.32±3.02	4.69±5.65	7.80±5.97	Placebo	
0.42	0.001	3.33±1.36	3.12±1.55	3.74±1.57	5.48±1.26	Intervention	DAS28-ESR
		3.34±1.18	3.59±1.21	4.07±1.39	5.66±1	Placebo	
0.42	0.001	22.96±20.90	22.59±21.76	38.14±25.27	62.51±29.30	Intervention	VAS
		15.20±15.57	24.40±21.22	34.40±24.16	66.80±27.19	Placebo	
0.42	0.001	0.74±0.6	0.83±0.67	0.94±0.64	1.36±0.67	Intervention	HAQ_DI
		0.52±0.41	0.68±0.64	0.72±0.67	1.48±0.69	Placebo	
0.49	0.001	0.85±0.75	0.71±0.53	1.21±0.85	2.07±0.77	Intervention	Pain Score
		0.52±0.42	0.87±0.75	0.80±0.75	2.27±0.88	Placebo	
0.89	0.001	2.22±1.84	3.70±7.78	3.48±2.04	5.87±2.10	Intervention	Physical function
		1.68±1.74	2.10±1.75	4.84±9.63	6.28±1.96	Placebo	
0.13	0.18	5.27±1.05	5.64±1.63	5.0	5.18±0.96	Intervention	Prednisolone (mg/day)
		5.0	5.0	5.0	5.19±0.98	Placebo	
0.49	0.06	8.31±2.81	8.51±2.52	7.68±1.18	7.59±0.48	Intervention	Methotrexate (mg/week)
		7.98±1.41	7.98±1.41	7.59±0.49	7.59±0.49	Placebo	

† P value between groups for independent T test for baseline. †† P value between groups for independent T test for 1 month follow up. ††† P value between groups for independent T test for 45 day follow up. †††† P value between groups for independent T test for 90 day follow up.

**Table 4:** Comparison of side effects between, saffron and placebo treated groups of RA patients

**Groups**	**Intervention**	**Placebo**	P value*
Xerostomia	12.2%	12.2%	0.99
Constipation	2.4%	7.3%	0.61
AUB	2.4%	2.4%	0.99
Palpitation	9.8%	9.8%	0.99
Restlessness	4.9%	14.6%	0.26
Anxiety	9.8%	9.8%	0.99
Nausea	4.9%	4.9%	0.99
Reflux	2.4%	4.9%	0.99
Abdominal pain	4.9%	2.4%	0.99
Headache	9.8%	4.9%	0.67
Dizziness	2.4%	4.9%	0.99
Vomiting	2.5%	2.4%	0.99
Paresthesia	4.9%	9.8%	0.67

AUB: Abnormal uterine bleeding. *p value from Fisher exact test.†Not reported in intervention group.

## Discussion

The present study evaluated the effectiveness of 100 mg pure saffron given as pills, in improving disease activity and quality of life in RA patients and assessed the quality of life by keeping the previous treatment regimen of the patients. The results suggested that prescribing saffron pills along with the standard therapy (prednisolone and methotrexate), in our RA patients did not change the disease activity (DAS-28-ESR) and quality of life (HAQ-DI) compared to the placebo group. Besides, intervention with saffron pills had no side effects, and there was no difference between these groups in terms of complications. Previous studies showed the antioxidant and anti-inflammatory effects of saffron in patients with RA (Amin and Hosseinzadeh, 2015 ▶;Hamidi et al., 2020b ▶; Moghimi et al., 2014b ▶; Rathore et al., 2015b ▶). It was also suggested that the aqueous extract of a saffron stalk, which mainly contains alkaloids and saponins, has an effect on pain and chronic inflammation (Hosseinzadeh and Younesi, 2002a ▶). According to the literature, saffron has many biological effects and is used as asedative, anticonvulsant, antidepressant, antispasmodic, menstrual enhancer, analgesic and sputum inducer, and is applied to induce sweating. Saffron could also help in treatment of scarlet fever, smallpox, colds, asthma, eye and heart disease, tumors, and cancer (Abdullaev 1993a ▶; Abdullaev 2002a ▶). Additionally, experimental studies using the aqueous extract of saffron flower in rats, indicated improved inflammation (Sahebari et al., 2011 ▶). In chronic inflammation, the aqueous and alcoholic extract as well as the alcoholic extract of the petal had an anti-inflammatory effect (Hosseinzadeh and Younesi, 2002 ▶). An experimental study by Sahebari et al. (2011) ▶ investigated the effects of aqueous extract of saffron flower in an animal model of arthritis. It was demonstrated that aqueous extract of saffron had a dramatic effect in reducing the width of foot and joint diameter of the ankle joint, arthritis index and motor restriction compared to the untreated group. Although, the results did not differ significantly from the dexamethasone group in this study, they were consistent with those of trial studies on the human.

In another study about the therapeutic effects of crocin (an effective ingredient of saffron) in rats with RA, Liu et al. (2018) ▶ observed that the clinical activity indicators and inflammatory and oxidative markers like IL-6, IL-17 and TNF-α in the crocin-treated group, had a significant decrease compared to the control group.

Hamidi et al. (2020) ▶ in an RCT investigated the effect of saffron supplement on clinical outcomes and metabolic profiles in patients with active RA. In their study, 66 women older than 18 years were divided into 2 groups; the intervention group received 100 mg/day saffron supplement (n=33) and the placebo group received matched placebo (n=33) a period of 12 weeks. The patients reported no adverse effect. Saffron supplementation significantly decreased the number of tender and swollen joints, pain intensity based on visual analogue, and disease activity score (DAS28) at the end of intervention between the two groups and in saffron group compared with the baseline values. Physician Global Assessment and erythrocyte sedimentation rate were significantly improved after intervention. At the end, high‐sensitivity C‐reactive protein reduced in intervention group compared with the baseline value. Tumor necrosis factor alpha, interferon gamma, and malondialdehyde were decreased, and total antioxidant capacity was increased, (but differences between groups were not significant). According to this study, saffron supplements could improve clinical outcomes in RA patients.

In contrast, our study suggested that there was not a notable difference in the ESR, disease activity or quality of life scores between the control and intervention groups, which can be attributed to the concurrent drug regimens in both groups.

Modaghegh and his colleague (2008) examined the safety of saffron pills in humans. They observed that high dose (400 mg) saffron decreased systolic and arterial blood pressure, reduced clinical criteria for CBC diff, hemoglobin and hematocrit and platelet count and increased sodium and blood urea nitrogen and creatinine. However, the present study did not show such differences between the mentioned groups, probably due to low dose (100 mg) saffron. 

Mansoori et al. (2011) ▶ also studied the safety of saffron and reported that consuming saffron in addition to a selective serotonin reuptake inhibitor, did not significantly change the laboratory parameters. The results of their study were consistent with those of the current study in terms of the side effects. While in the study of Gout et al. (2010) ▶, the incidence of side effects in those who were treated with a dose of 174.5 mg saffron was reported as 16%.

This study had some limitations and strengths. This study was performed on newly diagnosed patients who did not receive any other treatment before the intervention. During the three-month follow-up, there was not a significant change in the dosage of standard therapies in the groups.

The short-term duration of the study and choosing the minimum dose of saffron are the limitations of this study. Another limitation of this study was that smoking was not considered an exclusion criterion or a matching factor for categorizing the patients.

Generally, studies in animal models have indicated that high efficacy of saffron and crocin in reducing inflammatory and oxidative factors, while in human studies; there are not enough data to be able to conclude about the effectiveness of saffron supplementation. The exact duration of treatment with saffron required to achieve the best effect, the subtypes of saffron extract, and the best treatment dose of saffron should be investigated in detail in future. 

## References

[B1] Abdullaev F, Espinosa-Aguirre J (2004a). Biomedical properties of saffron and its potential use in cancer therapy and chemoprevention trials. Cancer detect prev.

[B2] Abdullaev FI (1993a). Biological effects of saffron. BioFactors (Oxford England).

[B3] Abdullaev FI (2002a). Cancer chemopreventive and tumoricidal properties of saffron (Crocus sativus L ). Expbiol med.

[B4] Amin B, Hosseinzadeh H (2012b). Evaluation of aqueous and ethanolic extracts of saffron, Crocus sativus L and its constituents, safranal and crocin inallodynia and hyperalgesia induced by chronic constriction injury model of neuropathic pain in rats. Fitoterapia.

[B5] Amin B, Hosseinzadeh H (2015). Analgesic and anti-inflammatory effects of Crocus sativus (saffron) Bioactive nutraceuticals and dietary supplements in neurological and brain disease. Elsevier.

[B6] Benucci M, Saviola G, Baiardi P, Manfredi M, Puttini PS, Atzeni F (2012). Determinants of risk infection during therapy with anti TNF-alpha blocking agents in rheumatoid arthritis. Open Rheumatol J.

[B7] Bolhassani A, Khavari A, Bathaie SZ (2014a). Saffron and natural carotenoids: Biochemical activities and anti-tumor effects. BiochimBiophysActa.

[B8] Bruce B, FriesJ F (2009a). The Health Assessment Questionnaire (HAQ)© and the Improved HAQ. Clin Exp Rheumatol.

[B9] Duesterdieck-Zellmer KF, Driscoll N, Ott JF (2012b). Concentration-dependent effects of tiludronate on equine articular cartilage explants incubated with and without interleukin-1β. Am J Vet Res.

[B10] Edwards C, Cooper C (2006a). Early environmental factors and rheumatoid arthritis. Clin Exp Immunol.

[B11] Fransen J, Creemers MCW, Van Riel PLCM (2004b). Remission in rheumatoid arthritis: agreement of the disease activity score (DAS28) with the ARA preliminary remission criteria. Rheumatology.

[B12] Ghorbani M (2008b). The efficiency of saffron’s marketing channel in Iran. World Appl Sci J.

[B13] Gout B, Bourges C, Paineau-Dubreuil S (2010b). Satiereal, a Crocus sativus L extract, reduces snacking and increases satiety in a randomized placebo-controlled study of mildly overweight, healthy women. Nutr Res.

[B14] Hamidi Z, Aryaeian N, Abolghasemi J (2020). The effect of saffron supplement on clinical outcomes and metabolic profiles in patients with active rheumatoid arthritis: A randomized, double-blind, placebo-controlled clinical trial. Phytother Res.

[B15] Hertzog M A ( 2008a). Considerations in determining sample size for pilot studies. Res Nurs Health.

[B16] Hosseinzadeh H (2014a). Saffron: a herbal medicine of third millennium. Jundishapur j nat pharm prod.

[B17] Hosseinzadeh H, Modaghegh MH, Saffari Z (2009b). Crocus sativus L (Saffron) extract and its active constituents (crocin and safranal) on ischemia-reperfusion in rat skeletal muscle. EVID-Based Compl Alt.

[B18] Hosseinzadeh H, Younesi HM (2002a). Antinociceptive and anti-inflammatory effects of Crocus sativus L stigma and petal extracts in mice. BMC PharmacolToxicol.

[B19] Hreggvidsdottir HS, Noordenbos T, Baeten DL (2014a). Inflammatory pathways in spondyloarthritis. Mol Immunol.

[B20] Hsu J, Chou F, Lee M (1999b). Suppression of the TPA-induced expression of nuclear-protooncogenes in mouse epidermis by crocetin via antioxidant activity. Anticancer Res.

[B21] Javadi B, Sahebkar A, Emami SA (2013a). A survey on saffron in major Islamic traditional medicine books. Iran J Basic Med Sci.

[B22] Kasper D, Harrison TR (2005). Harrison's principles of internal medicine.

[B23] Kay J, Upchurch KS ( 2012). ACR/EULAR 2010 rheumatoid arthritis classificationcriteria. Rheumatology.

[B24] Korani S, Korani M, Sathyapalan T, Sahebkar A (2019). Therapeutic effects of Crocin in autoimmune diseases: A review. BioFactors.

[B25] Lee I-A, Lee JH, Baek N-I, Kim D-H (2005b). Antihyperlipidemic effect of crocin isolated from the fructus of Gardenia jasminoides and its metabolite crocetin. Biol Pharm Bull.

[B26] Li X, Jiang C, Zhu W (2017b). Crocin reduces the inflammation response in rheumatoid arthritis. Biosci Biotech Bioch.

[B27] Liu W, Sun Y, Cheng Z, Guo Y, Liu P, Wen Y (2018a). Crocin exerts anti-inflammatory and anti-arthritic effects on type II collagen-induced arthritis in rats. Pharm Biol.

[B28] Mansoori P, Akhondzadeh S, Raisi F, Ghaeli P (2011). A randomized, double-blind, placebo-controlled study of safety of the adjunctive saffron on sexual dysfunction induced by a selective serotonin reuptake inhibitor. J Med Plant Res.

[B29] Modaghegh M-H, Shahabian M, Esmaeili H-A, Rajbai O (2008). Safety evaluation of saffron (Crocus sativus) tablets in healthy volunteers. Phytomedicine.

[B30] Moghimi N, Rahimi E, Ghaderi B, Saeeedi A (2014b). Relationship between platelet indices and severity of rheumatoid arthritis according to DAS28 criteria. Scientific J Kurdistan Univ Med Sci.

[B31] Molina V, Shoenfeld Y (2005b). Infection, vaccines and other environmental triggers of autoimmunity. J Autoimmun.

[B32] Nair SC, Kurumboor S, Hasegawa J (1995b). Saffron chemoprevention in biology and medicine: a review. Cancer Biother Radiopharm.

[B33] Nazary-Moghadam S, Zeinalzadeh A, Salavati M, Almasi S, Negahban H (2017). Adaptation, reliability and validity testing of a Persian version of the Health Assessment Questionnaire-Disability Index in Iranian patients with rheumatoid arthritis. J J Body Mov Ther.

[B34] NG KP, AUSTIN P, AMERATUNGA R, McQUEEN F (2006b). Role of anticycliccitrullinated peptide 2 assay in long‐standing rheumatoid arthritis. APLAR J Rheumatol.

[B35] Padyukov L, Silva C, Stolt P, Alfredsson L (2004). A gene–environment interaction between smoking and shared epitope genes in HLA–DR provides a high risk of seropositive rheumatoid arthritis. Arthritis Rheum.

[B36] Rastmanesh R, Rabiee S, Shaabani Y, Mazinani H, Ebrahimi AA, Jamshidi AR (2010b). Validation of the Persian version of the Stanford Health Assessment Questionnaire (HAQ) in patients with rheumatoid arthritis. J Paramed Sci.

[B37] Rathore B, Jaggi K, Thakur SK, Mathur A (2015b). Anti-inflammatory activity of Crocus sativus extract in experimental arthritis. Int J Pharm Sci Res.

[B38] Sahebari M, Mahmoudi Z, Rabe SZT, Haghmorad D (2011a). Inhibitory effect of aqueous extract of Saffron (Crocus sativus L ) on adjuvant-induced arthritis in Wistar rat. Pharmacologyonline.

[B39] Schmidt M, Betti G, Hensel A (2007). Saffron in phytotherapy: pharmacology and clinical uses. Wien Med Wochenschr.

[B40] Tremblay I, Beaulieu Y, Bernier A, Crombez G, Laliberté S, Thibault P, Velly AM, Sullivan MJ (2008). Pain catastrophizing scale for francophone adolescents: a preliminary validation. Pain Res Manag.

[B41] Tobón GJ, Youinou P, Saraux A (2010b). The environment, geo-epidemiology, and autoimmune disease: Rheumatoid arthritis. Autoimmun Rev.

[B42] Van Der Heijde D, Van Der Helm-Van, AH, Aletaha D, Bingham CO, Burmester GR, Dougados M, Emery P, Felson D, Knevel R, Kvien TK, Landewé RB (2013a). EULAR definition of erosive disease in light of the 2010 ACR/EULAR rheumatoid arthritis classification criteria. Ann Rheum Dis.

[B43] Yarijani ZM, Najafi H, Madani SH (2016b). Protective effect of crocin on gentamicin-induced nephrotoxicity in rats. Iran J Basic Med Sci.

[B44] Zamani Taghizadeh Rabe S, Sahebari M, Mahmoudi Z, Hosseinzadeh H (2015b). Inhibitory effect of Crocus sativus L ethanol extract on adjuvant-induced arthritis. Food Agr Immunol.

